# Impact of PD-L1 and PD-1 Expression on the Prognostic Significance of CD8^+^ Tumor-Infiltrating Lymphocytes in Non-Small Cell Lung Cancer

**DOI:** 10.3389/fimmu.2021.680973

**Published:** 2021-05-26

**Authors:** Enrico Munari, Marcella Marconi, Giulia Querzoli, Gianluigi Lunardi, Pietro Bertoglio, Francesco Ciompi, Alice Tosadori, Albino Eccher, Nicola Tumino, Linda Quatrini, Paola Vacca, Giulio Rossi, Alberto Cavazza, Guido Martignoni, Matteo Brunelli, George J. Netto, Lorenzo Moretta, Giuseppe Zamboni, Giuseppe Bogina

**Affiliations:** ^1^ Pathology Unit, IRCCS Sacro Cuore Don Calabria, Negrar di Valpolicella, Italy; ^2^ Department of Molecular and Translational Medicine, University of Brescia, Brescia, Italy; ^3^ Clinical Analysis Laboratory and Transfusional Medicine, IRCCS Sacro Cuore Don Calabria, Negrar di Valpolicella, Italy; ^4^ Division of Thoracic Surgery, IRCCS Maggiore Teaching Hospital and Sant’Orsola University Hospital, Bologna, Italy; ^5^ Computational Pathology Group, Department of Pathology, Radboud University Medical Center, Nijmegen, Netherlands; ^6^ Pathology Unit, University and Hospital Trust of Verona, Verona, Italy; ^7^ Immunology Area, Bambino Gesù Children’s Hospital (IRCCS), Rome, Italy; ^8^ Pathology Unit, AUSL della Romagna, Ravenna, Italy; ^9^ Pathology Unit, AUSL/IRCCS of Reggio Emilia, Reggio Emilia, Italy; ^10^ Department of Diagnostics and Public Health, University of Verona, Verona, Italy; ^11^ Pathology Unit, Pederzoli Hospital, Peschiera del Garda, Italy; ^12^ Department of Pathology, University of Alabama at Birmingham, Birmingham, AL, United States

**Keywords:** PD-L1, PD-1, lung cancer, immunoscore, CD8, TILs, prognosis

## Abstract

The immune infiltrate within tumors has proved to be very powerful in the prognostic stratification of patients and much attention is also being paid towards its predictive value. In this work we therefore aimed at clarifying the significance and impact of PD-L1 and PD-1 expression on the prognostic value of CD8^+^ tumor infiltrating lymphocytes (TILs) in a cohort of consecutive patients with primary resected non-small cell lung cancer (NSCLC). Tissue microarrays (TMA) were built using one representative formalin fixed paraffin embedded block for every case, with 5 cores for each block. TMA sections were stained with PD-L1 (clone SP263), PD-1 (clone NAT105) and CD8 (clone SP57). Number of CD8^+^ cells per mm^2^ were automatically counted; median, 25^th^ and 75^th^ percentiles of CD8^+^ cells were used as threshold for statistical clinical outcome analysis and evaluated in patients subgroups defined by expression of PD-L1 and PD-1 within tumors. We found an overall strong prognostic value of CD8^+^ cells in our cohort of 314 resected NSCLC, especially in PD-L1 negative tumors lacking PD-1^+^ TILs, and demonstrated that in PD-L1 positive tumors a higher density of CD8^+^ lymphocytes is necessary to improve the prognosis. Our data strengthen the concept of the importance of the assessment and quantification of the immune contexture in cancer and, similarly to what has been carried on in colorectal cancer, promote the efforts for the establishment of an Immunoscore for NSCLC for prognostic and possibly predictive purposes.

## Introduction

Traditionally, tumor staging has always been performed according to the evaluation of the pathological features defined in the American Joint Committee on Cancer/Union for International Cancer Control (AJCC/UICC) tumor-node-metastasis (TNM) system (1).

However, the concurrent evaluation of the immune infiltrate within tumors has proved to be very powerful in the stratification of patients within different prognostic groups with higher precision. As a matter of fact, the “Immunoscore” has reached an advanced stage of development in colon cancer, where the evaluation of the immune infiltrate, namely CD3^+^ and CD8^+^ lymphocytes, has been demonstrated to be an important additional parameter to be integrated with the TNM (2). In this regard, multicenter prospective studies have been undertaken by an international task force with the aim to further implement the use of the Immunoscore with the TNM (designated TNM-I) in clinical practice (3). A similar effort has been promoted for the evaluation of the role of tumor infiltrating lymphocytes (TILs) in breast cancer (4). In non-small cell lung cancer, different studies have evaluated the prognostic impact of TILs using different approaches and methods in terms of types of cells, compartment, scoring and material (5).

Along with the growing awareness of the importance of the immune infiltrate as a variable for better prognostic stratification, much attention is being paid towards its predictive value. Immunotherapy targeting the PD-1/PD-L1 axis has shown remarkable efficacy in different tumor types and has become the standard of care for the management of locally advanced and metastatic NSCLC (6–9). Despite the great promise held by PD-1/PD-L1 pathway inhibitors, in clinical trials, only a fraction of unselected patients with advanced NSCLC showed sustained response (6, 7). Thus, the selection of patients with the highest chance of response is critical; however, so far, only the evaluation of PD-L1 expression has been approved for guiding treatment decisions for anti PD-1/PD-L1 therapy (8, 9). Strategies for implementing the predictive potential of PD-L1 testing are therefore urgently needed. In this regard, a better understanding of the interaction between the PD-1/PD-L1 axis and tumor-infiltrating lymphocytes is of critical importance. In this work we therefore aimed at clarifying such interaction by assessing the significance and impact of PD-L1 and PD-1 expression on the prognostic value of CD8^+^ TILs in NSCLC.

## Methods

### Patients

The study cohort consisted of consecutive patients with primary NSCLC who had undergone surgical resection at the IRCCS Sacro Cuore Don Calabria Hospital of Negrar, Verona, Italy, between 2003 and 2018 and for whom slides and paraffin-embedded tissue blocks were available. None of the patients received neoadjuvant chemotherapy or radiotherapy prior to thoracic surgery. Tumors were classified according to the 2015 WHO classification, and staging was done by using the TNM staging manual (eight edition) (1). Patient demographics and clinical data were retrieved from the institution’s digital archives. The investigations were conducted according to the principles expressed in the Declaration of Helsinki

### Tissue Samples and Immunohistochemistry (IHC)

For each case, all hematoxylin and eosin–stained slides were reviewed for confirmation of diagnosis; one block was then selected for tissue microarrays (TMAs) construction. For each block, five cores with a diameter of 1 mm were obtained from diverse areas of the tumor and randomly numbered from 1 to 5. From each TMA 5-μm sections were cut and stained with CD8 (clone SP57, Ventana Medical Systems, Tucson, AZ), PD-1 (clone NAT105, Roche, Basel, Switzerland) and PD-L1 (clone SP263, Roche, Basel, Switzerland) on an automated staining platform (Benchmark Ultra [Ventana Medical Systems]). An OptiView DAB IHC Detection Kit (Ventana Medical Systems) and an OptiView Amplification Kit (Ventana Medical Systems) were used according to the manufacturer’s recommendations for visualization of the primary anti-CD8, anti-PD-1 and anti–PD-L1 antibodies. Stained sections were scanned with a Ventana iScan HT slide scanner (Ventana Medical Systems). PD-L1 expression was evaluated independently by two pathologists (GB and GR) and calculated as the percentage of tumor cells with membrane staining of any intensity for each core; the final score was calculated as the average of all available cores. Cases were considered positive for PD-L1 when ≥1% of the tumor cells expressed PD-L1. Discordant cases were re-evaluated by both pathologist for consensus. PD-1 was evaluated by two pathologists (EM and GQ) and scored as the percentage of positive immune cells for each core, using the median as cut-off value for each case. Concerning CD8, the absolute numbers of CD8-positive cells per mm^2^ were automatically counted using QuPath version 0.2.0 (10). The cut-off value for CD8 expression was determined as the median absolute number. Subsequently, the 25^th^ and 75^th^ percentiles absolute number were used as threshold for statistical clinical outcome analysis.

### Statistical Analysis

Data were imported and analyzed using STATA/IC for windows version 14.0.

Chi-square tests were used to statistically analyze the association between CD8 positive cells, clinicopathological variables and PD-L1 and PD-1 expression.

Disease-free survival (DFS) was calculated from the date of surgery to the date of recurrence or the date of death from any cause. Overall survival (OS) was calculated from the date of surgery to the date of death from any cause. For stage IV patients OS only was considered. Patients alive and not relapsing or alive regardless of relapsing were censored at the time of their last follow-up visit for DFS and OS, respectively. Long-term survivors were censored at 120 months of follow-up. Cumulative incidence of DFS and OS in the groups was described by the Kaplan–Meier method and compared with the log-rank test.

The Cox proportional hazard regression model was used to evaluate the associations between clinicopathological factors (sex, age, histology, surgery, stage, therapy) and clinical outcome.

A two-sided P value <0.05 was considered statistically significant.

## Results

### Clinico-Pathologic Features

Overall, 314 patients with tissue-confirmed resected NSCLC were included in this study. The median age at diagnosis was 70 (range 40–86) years and 221 (70.4%) patients were men. The most prevalent histology was adenocarcinoma (71.4%). The majority of patients underwent lobectomy (77.1%) and had stage I/II disease (76.1%). Adjuvant treatment, consisting of chemotherapy and/or radiotherapy, was performed in 44 patients; none of the patients received immunotherapy. PD-L1 expression was seen in 106 of 314 NSCLC (33.8%). The mean and median number of CD8-positive cells were 750 (SD: 635) and 575 (range: 10–3015) per mm^2^, respectively. The cut-off value for CD8 expression was determined as the median absolute number and cases were considered positive when CD8-positive cells ≥575 per mm^2^ were observed. The clinicopathologic features based on CD8 median expression are shown in **Table 1**, where it can be seen a clear association between amount of CD8 lymphocytes and PD-L1 expression on tumors cells, as well as a positive correlation with PD-1 expression (analysis possible for 297 cases). Indeed, PD-L1 expression in tumor cells and PD-1 expression in TILs showed a positive correlation (**Supplementary Table 1**). The median number of CD8 positive lymphocytes, in the PD-L1 negative and positive tumors, was 505 and 813 per mm^2^ respectively. Considering this different amount of CD8^+^ lymphocytes in the PD-L1 negative and positive subgroups, the DFS and OS analysis was performed using both the median (575 per mm^2^) and the first (300 per mm^2^) and third (950 per mm^2^) quartiles as the threshold.

**Table 1:** CD8^+^ TILs in 314 NSCLC in relation to clinicopathologic variables.

	Overall (%)	CD8^+^ TILs		P value
		< 575 x mm² (%)	≥ 575 x mm² (%)	
**Patients**	314	154	160	
**Sex**				
Male	221 (70.4)	100 (45.3)	121 (54.7)	***0.03***
Female	93 (29.6)	54 (58.1)	39 (41.9)	
**Age** (years)				
≤70	163 (51.9)	79 (48.5)	84 (51.5)	*0.83*
>70	151 (48.1)	75 (49.7)	76 (50.3)	
**Histology**				
Adenocarcinoma	224 (71.4)	107 (48.5)	84 (51.5)	*0.73*
SCC	72 (22.9)	37 (51.4)	35 (48.6)	
Others	18 (5.7)	10 (55.6)	8 (44.4)	
**Surgery**				
Wedge/Segmentectomy	56 (17.8)	24 (42.8)	32 (57.2)	*0.51*
Lobectomy	244 (77.7)	122 (50)	122 (50)	
Pneumonectomy	14 (4.5)	8 (57.1)	6 (42.9)	
**TNM Stage**				
I	159 (50.6)	77 (48.4)	82 (51.6)	*0.30*
II	80 (25.5)	45 (56.2)	35 (43.8)	
III	53 (16.9)	26 (49.1)	27 (50.9)	
IV	16 (5.1)	5 (31.3)	11 (68.7)	
Unknow	6 (1.9)	1	5	
**Adjuvant Treatment**				
No	242 (77.1)	144 (47.1)	128 (52.9)	*0.14*
Yes	44 (14.0)	26 (59.1)	18 (40.9)	
Unknow	28 (8.9)	14	14	
**PD-L1 (1% threshold)**				
Negative	208 (66.2)	122 (58.7)	86 (41.3)	***0.00***
Positive	106 (33.8)	32 (30.2)	74 (69.8)	
**PD-1 (7.5% threshold)**				
Negative	143 (45.6)	92 (59.7)	51 (31.9)	***0.00***
Positive	154 (49.0)	56 (36.4)	98 (61.2)	
Unknow	17 (5.4)	6 (3.9)	11 (6.9)	

There were no other significant differences in patients’ characteristics between groups based upon CD8 median expression except for gender.

### Clinical Outcome

In the entire cohort, disease progression analysis could be performed in 234 patients: in 63 patients follow-up was unknown, while 16 were excluded because had distant metastases at the time of diagnosis. Disease progression occurred in 90 patients: 33 experienced loco-regional recurrence and 57 distant metastases. The median relapsing time was 12 months (95% C.I. 10-16). Overall survival analysis was possible in 293 patients: 120 patients were dead at the time of analysis. The median time to death was 27 months (95% C.I. 23-30). Patients alive and disease-free at the time of analysis had a median follow-up of 50 months (95% C.I. 41-56).

#### Survival Analysis According to CD8^+^ TILs Density

**Figure 1:**
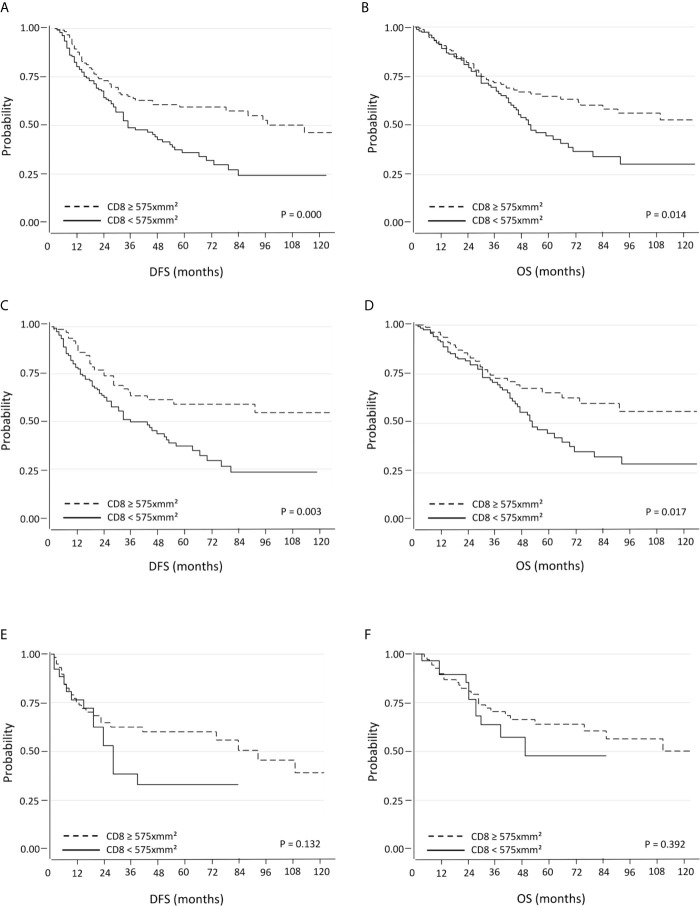
Kaplan-Meier curves for DFS and OS based on CD8^+^ TILs using the median as the threshold in all **(A, B)**, in PD-L1 negative **(C, D)** and PD-L1 positive **(E, F)** NSCLC.

The group of patients whose tumors showed high CD8^+^ cell density (≥575 per mm^2^) showed a significant increase in DFS (median 34.5 months; 95% C.I. 26-47) compared to the group with low CD8^+^ cell density (<575 per mm^2^) (median DFS: 25 months; 95% C.I. 20-29; p=0.000) (**Figure 1A**). OS was also significantly increased in high CD8^+^ cell density group (median 41 months; 95% C.I. 37-49) as compared with the low CD8^+^ cell density group (median 32.5 months; 95% C.I. 28-37; p=0.014) (**Figure 1B**).

Multivariate Cox proportional hazards regression analysis showed that both high density of CD8^+^ cells and stage I were favorable prognostic factor for DFS and OS while female sex was associated with significantly better OS (**Table 2A**).

**Table 2:** Multivariate analysis of predictors of DFS and OS in all NSCLC **(A)** and in PD-L1 negative NSCLC **(B)** using the median of CD8^+^ TILs as the threshold.

Variables	DFS			OS		
	HR	95% CI	P value	HR	95% CI	P value
**A**
**Sex**
Male	1			1		
Female	0.73	0.47-1.12	0.15	0.62	0.39-1.00	0.05
**Age** (years)
≤70	1			1		
>70	0.94	0.62-1.42	0.78	1.08	0.69-1.07	0.71
**Histology**
Adenocarcinoma	1			1		
Others	0.86	0.56-1.31	0.49	0.86	0.55-1.34	0.51
**Surgery**
Wedge/Lobectomy	1			1		
Pneumonectomy	0.57	0.25-1.27	0.17	0.76	0.34-1.69	0.50
**AdJuvant Treatment**
No	1			1		
Yes	1.00	0.58-1.73	0.97	1.14	0.64-2.01	0.65
**TNM Stage**
II - IV	1			1		
I	0.27	0.17-0.43	**0.00**	0.31	0.19-0.50	**0.00**
**CD8**
<575/mm²	1			1		
≥575/mm²	0.49	0.33-0.73	**0.00**	0.57	0.38-0.87	**0.00**
**B**
**Sex**
Male	1			1		
Female	0.64	0.39-1.06	0.08	0.57	0.33-099	**0.04**
**Age** (years)
≤70	1			1		
>70	1.07	0.65-1.76	0.76	1.24	0.73-2.11	0.42
**Histology**
Adenocarcinoma	1			1		
Others	0.85	0.48-1.50	0.59	0.67	0.36-1.24	0.21
**Surgery**
Wedge/Lobectomy	1			1		
Pneumonectomy	0.36	0.10-1.23	0.10	0.63	0.18-2.14	0.46
**AdJuvant Treatment**
No	1			1		
Yes	1.01	0.51-1.96	0.97	1.08	0.53-2.18	0.82
**TNM Stage**
II - IV	1			1		
I	0.25	0.14-0.44	**0.00**	0.30	0.17-0.53	**0.00**
**CD8**
<575/mm²	1			1		
≥575/mm²	0.43	0.25-0.73	**0.00**	0.49	0.28-0.83	**0.00**

Higher CD8 TILs density was associated with better DFS also using both 25^th^ (300 per mm^2^) and 75^th^ (950 per mm^2^) percentile cut-off (**Supplementary Figures 1A, C**).

#### Survival Analysis According to PD-L1 and PD-1 Expression

PD-L1 expression was associated neither with DFS (p=0.862) nor with OS (p=0.882), even after subgroup analysis according to low CD8^+^ TILs density (DFS: p=0.741; OS: p=0.853) or high CD8^+^ TILs density (DFS: p=0.346; OS: p=0.467).

PD-1 expression was associated with neither DFS (p=0.114) nor OS (p=0.142) when considering the entire cohort.

#### Survival Analysis According to CD8^+^ TILs Density Using the Median (575 CD8^+^ Cells per mm^2^) as the Threshold and PD-L1 and PD-1 Expression

When patients with PD-L1 negative tumors were considered, high density of CD8^+^ cells was associated with a significantly better DFS (median 37 months; 95% C.I. 26-50) and OS (median 45 months; 95% C.I. 37-52) compared to those with low CD8^+^ cells density (median DFS: 25.5 months, 95% C.I. 20-30, p=0.003; median OS: 34 months, 95% C.I. 30-39, p=0.017) (**Figures 1C , D**). The positive prognostic value of high CD8^+^ cell density was also maintained in multivariate analysis (**Table 2B**). Better DFS and OS was demonstrated also for patients with tumors with high CD8^+^ cells density lacking PD-1 expression, but not for cases with PD-1^+^ TILs (**Supplementary Figures 2A, B**). An even bigger difference in both DFS (p=0.000) and OS (0.003) emerged for patients with PD-L1 negative tumors with high density of CD8^+^ TILs lacking PD-1 expression **(** **Supplementary Figure 3** **).**

Conversely, there were not significant differences in DFS and OS within the of PD-L1 positive tumors subgroup (**Figures 1E, F**).

#### Survival Analysis According to PD-L1 Expression and CD8^+^ TILs Density Using the 25^th^ Percentile (300 CD8^+^ Cells per mm^2^) as the Threshold

**Figure 2:**
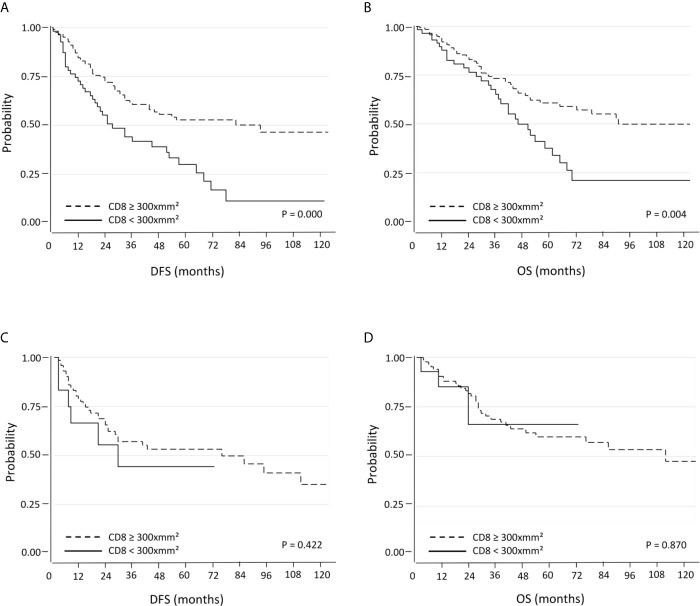
Kaplan-Meier curves for DFS and OS based on CD8^+^ TILs using the 25^th^ percentile as the threshold in PD-L1 negative **(A, B)** and PD-L1 positive **(C, D)** NSCLC.

In this setting the results are superimposable to those observed previously, where the CD8 median was used as threshold. Within the PD-L1 negative tumors subgroup, patients with tumors with high CD8^+^ cell density showed significantly better DFS (median 32 months; 95% C.I. 26-39) and OS (median 40.5 months; 95% C.I. 33-46) compared to those with low CD8^+^ cell density (median DFS: 20 months, 95% C.I. 17-30, p=0.000; median OS: 35 months, 95% C.I. 23-41, p=0.004) (**Figures 2A, B**). The positive prognostic value of CD8 expression was also maintained in multivariate analysis (**Table 3A**). Conversely, there were not significant differences in terms of DFS and OS when PD-L1 positive tumors were considered (**Figures 2C, D**).

**Table 3:** Multivariate analysis of predictors of DFS and OS in PD-L1 negative NSCLC using the 25^th^ percentile of CD8^+^ TILs **(A)** and in PD-L1 positive NSCLC using the 75^th^ percentile of CD8^+^ TILs as threshold **(B)**.

Variables	DFS			OS		
	HR	95% CI	P value	HR	95% CI	P value
**A**						
**Sex**
Male	1			1		
Female	0.62	0.38-1.02	0.06	0.57	0.33-0.98	**0.04**
**Age** (years)
≤70	1			1		
>70	1.20	0.74-1.97	0.45	1.25	0.74-2.12	0.40
**Histology**
Adenocarcinoma	1			1		
Others	0.95	0.55-1.66	0.87	0.70	0.38-1.28	0.24
**Surgery**
Wedge/Lobectomy	1			1		
Pneumonectomy	0.36	0.11-1.20	0.09	0.64	0.19-2.16	0.48
**AdJuvant Treatment**
No	1			1		
Yes	1.04	0.54-1.20	0.91	1.12	0.56-2.23	0.74
**TNM Stage**
II - IV	1			1		
I	0.28	0.16-0.49	**0.00**	0.31	0.17-0.55	**0.00**
**CD8**
<300/mm²	1			1
≥300/mm²	0.48	0.30-0.77	**0.00**	0.50	0.31-0.82	**0.00**
**B**						
**Sex**
Male	1			1		
Female	0.91	0.38-2.21	0.84	0.66	0.24-1.83	0.43
**Age** (years)
≤70	1			1		
>70	0.54	0.22-1.30	0.17	0.81	0.33-2.03	0.66
**Histology**
Adenocarcinoma	1			1		
Others	1.01	0.51-2.00	0.51	1.25	0.62-2.54	0.52
**Surgery**
Wedge/Lobectomy	1			1		
Pneumonectomy	1.02	0.34-3.10	0.96	0.96	0.32-2.92	0.95
**AdJuvant Treatment**
No	1			1		
Yes	0.60	0.19-1.87	0.19	0.95	0.30-2.91	0.92
**TNM Stage**
II - IV	1			1		
I	0.28	0.11-0.72	**0.00**	0.32	0.12-0.82	**0.02**
**CD8**
<950/mm²	1			1		
≥950/mm²	0.28	0.12-0.65	**0.00**	0.47	0.21-1.03	0.06

#### Survival Analysis According to PD-L1 Expression and CD8^+^ TILs Density Using the 75^th^ Percentile (950 CD8^+^ Cells per mm^2^) as the Threshold

**Figure 3:**
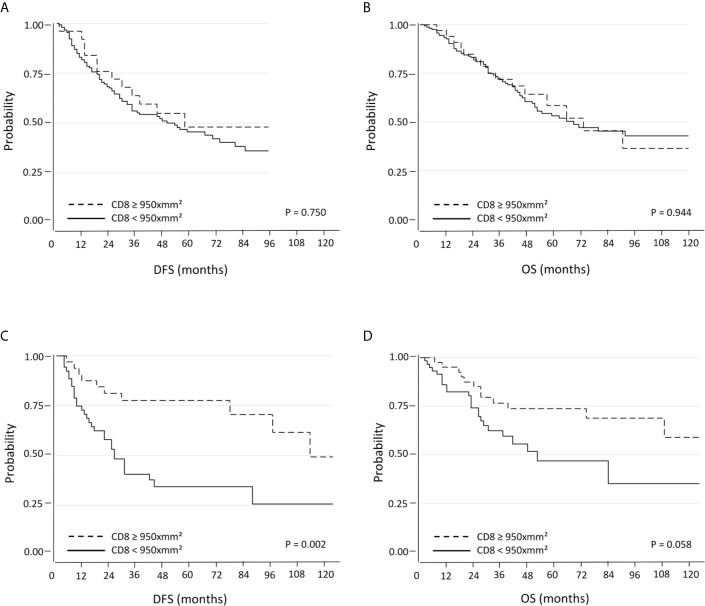
Kaplan-Meier curves for DFS and OS based on CD8^+^ TILs using the 75^th^ percentile as the threshold in PD-L1 negative **(A, B)** and PD-L1 positive **(C, D)** NSCLC.

Here the results are opposite to the two previous settings. When patients with PD-L1 negative tumors were considered, there were no significant differences in terms DFS and OS with respect to CD8^+^ TILs density (**Figures 3A, B**). On the other hand, PD-L1 positive tumors with high CD8^+^ TILs density were associated with a significantly longer DFS (median 52.5 months; 95% C.I. 27-70) compared with low CD8^+^ TILs density (median 20 months; 95% C.I. 17-30; p=0.002). Regarding OS, there was also a trend towards a statistically significant difference between high CD8^+^ TILs density (median 52.5 months; 95% C.I. 39-73) and low CD8^+^ TILs density (median 27 months; 95% C.I. 23-36; p=0.058) (**Figures 3C, D**). The positive prognostic value of high CD8^+^ cell TILs density was also maintained in multivariate analysis (**Table 3B**).

## Discussion

Our study confirms the overall strong prognostic value of CD8^+^ TILs in NSCLC using a digital approach for automatic absolute quantification of CD8^+^ cell density and provide evidence that in PD-L1 positive tumors, a higher density of CD8^+^ lymphocytes is necessary to improve the prognosis. In other words, if tumors do not express PD-L1, even few CD8^+^ cells can exert their antitumor effect; on the contrary, if tumors do express PD-L1, a higher number of CD8^+^ TILs is required in order to improve survival. Moreover, the presence of an efficient population of cytotoxic cells with low PD-1 expression is associated with prolonged DFS and OS, especially within tumors lacking PD-L1.

Traditional tumor staging schemes are based on the TNM classification in order to evaluate the extent of cancer spread, estimate patient outcome and guide therapeutic approaches for a variety of tumor types (1). However, significant differences exist between patients within the same pathological stages, underlying the limitations of the TNM system. Different parameters have been thus taking into consideration to refine cancer classification, including tumor immunophenotype, molecular and genetic features, often underestimating the important role of tumor microenvironment.

The last years have witnessed a paradigm shift from a tumor-centric view, mostly focused at detecting tumor cell features through the extensive use of “omics” approaches, to a more comprehensive consideration of the tumor microenvironment (TME) and immune components. Indeed, understanding the critical role of the antitumor activity of both innate and adaptive immune system for patient’s survival and the development of the concept of “immune contexture” represent important advances in oncology (11). As a matter of fact, in recent years, many efforts have been made in order to assess the prognostic impact of multiple immune cell types within the microenvironment of different tumors. Among the most important achievements in this field of investigation is the immunoscore, an assay based on the digital quantification of CD3^+^ and CD8^+^ lymphocytes both at the edge and at the center of tumors, which provides a scoring system defined as low or high in both locations. Such combination proved to be more precise than the TNM in predicting disease free survival, disease specific survival ad overall survival in stage I, II and III colorectal cancer (3, 12).

In general, besides the combination of CD3^+^ ad CD8^+^ lymphocytes, it appears that the TILs subtype with the strongest positive prognostic impact is represented by CD8+ cytotoxic T cells, as demonstrated across several cancer types (11, 13). Also in lung cancer different studies have assessed the prognostic impact of TILs with different methodologies with regards to type of cell evaluated, compartment (stroma and/or intraepithelial cells), scoring (continuous vs semiquantitative), quantification (manual vs digital) and type of material (tissue microarrays vs whole sections); overall it appears that CD8^+^ cells are the most promising, regardless of the compartment analyzed, with some variation in the statistical trends (5). Although studies have demonstrated high concordance between manual and digital quantification (14), digital imaging may improve reproducibility in the assessment of TILs compared with visual semiquantitative methods, and is advocated by the authors of the immunoscore (2). We have therefore chosen a digital approach for automatic absolute quantification of CD8^+^ cell density, using tissue microarrays. PD-L1 was instead evaluated visually as recommended by guidelines while PD-1 was defined as the percentage of positive TILs. Considering the median of CD8^+^ cell density as the threshold, we found a significant improvement in terms of DFS and OS for patients with tumors with high CD8^+^ cells density compared with low CD8^+^ cells density and such difference proved to be significant in multivariate analysis. This result is in line with what reported by Donnem et al. (15). These authors evaluated stromal CD8^+^ lymphocytes in a total of 797 NSCLC using a three-tiered approach based on manual evaluation of the percentage of CD8^+^ cells over total amount of nucleated cells. These authors found that stromal CD8^+^ TILs density has independent prognostic value in resected NSCLC in all endpoints (DFS, DSS and OS). At variance with this study, we used an absolute count of CD8^+^ cells per mm^2^ using an open-source software (10). Such method, in our opinion, eliminates interobserver variability and has the advantage of being quicker and more reproducible. We wanted to use the simplest method to define the threshold and decided to use the median of CD8^+^ cells as the cutoff, which resulted to be 575 cells per mm^2^. The same approach has been used by Kim et al. (16), who evaluated both CD8^+^ cells and PD-L1 expression in a smaller cohort of resected NSCLC patients. These authors found that the combination of high density of CD8^+^ cells and negative expression of PD-L1 was associated with a much better prognosis compared to all other CD8/PD-L1 combinations in terms of both OS and relapse free survival (RFS). We validated such observations on a larger cohort of patients and demonstrated that the prognostic significance of CD8^+^ cells is significantly influenced by PD-L1 expression in NSCLC. Indeed, when patients with PD-L1-negative tumors were considered, a significant independent prognostic impact of CD8^+^ TILs was observed using the first quartile and the median as the thresholds. Better DFS and OS was demonstrated also for patients with tumors with high CD8^+^ cells density lacking PD-1 expression, but not for cases with PD-1^+^ TILs. Moreover, an even bigger difference in both DFS and OS emerged for patients with PD-L1 negative tumors with high density of CD8+ TILs lacking PD-1 expression. On the contrary, for patients with PD-L1-positive tumors, CD8^+^ TILs were prognostically significant when the third quartile was considered as the cutoff. A logical explanation is that the presence of PD-L1 on tumor cells may contribute to tumor immune evasion by inhibiting cytotoxic lymphocytes, and only when they are in high number, they can exert their antitumor effect. On the other hand, both CD4^+^ and CD8^+^ lymphocytes, as well as NK cells, are known to induce PD-L1 expression on tumor cells through an IFN-γ mediated mechanism (17, 18); therefore, it might be possible that an immune-rich TME could in turn induce PD-L1 expression. In this setting, it is reasonable to believe that a tumor expressing PD-L1 and with a dense infiltrate of CD8^+^ TILs may respond better to PD1/PD-L1 inhibitors. However, evaluation of PD-L1 expression on tumor cells is currently the only biomarker approved to select patients for treatment with PD1/PD-L1 axis inhibitors, with several limitations due to tumor heterogeneity (19), differences between clones (20) and types of material (21). Thus, PD-L1 positivity by itself does not appear to be sufficiently accurate at present and therefore additional biomarkers for better prediction of clinical response to anti PD-1/PD-L1 antibodies are urgently needed. In this regard, Fumet et al. demonstrated that high expression of CD8^+^ in TILs evaluated with both immunohistochemistry and mRNA quantification was significantly associated with response rate and progression free survival in a cohort of 85 patients treated with nivolumab in second or third line. Importantly, these authors reported that high mRNA expression of both CD8 and PD-L1 outperformed the discriminatory properties of CD8 or PD-L1 alone, both at immunohistochemical and mRNA expression level (22). In another paper, Mazzaschi et al. demonstrated that resected NSCLC tumors highly infiltrated by CD8^+^ TILs with low PD-1 expression showed improved progression free survival in patients treated with nivolumab (23). Overall, it appears that understanding the immune contexture within tumors would not only improve the prognostic stratification of patients but could effectively play a major role in treatment decision, refining prediction of response to PD-1/PD-L1 axis inhibitors and future immunomodulatory agents. It is conceivable that in the foreseeable future, parameters relative to the immune contexture will gain more and more importance across different cancer types and will be included into the TNM staging system. The immunoscore developed for colon cancer proved to be an important example of a simple yet effective approach for the assessment of the immune microenvironment of tumors that is now approved for clinical use as an *in vitro* diagnostic test for colorectal cancer (24, 25).

In conclusion, in this study we added further data to support the notion that CD8^+^ TILs represent an efficient tool to refine prognostic stratification in NSCLC, for which we advocate the use of digital counting as a reproducible and less time-consuming method for quantification. Moreover, we demonstrated that PD-L1 expression has an impact on the prognostic value of CD8^+^ cells density, since its presence on tumor cells requires a higher density of CD8^+^ lymphocytes in order for them to exert antitumor function and therefore enhance patient survival. An even stronger prognostic impact for CD8+ cells was demonstrated in PD-L1 negative tumors lacking PD-1+ TILs. Our data strengthen the concept of the importance of the assessment and quantification of the immune contexture in cancer and, similarly to what has been carried on in colorectal cancer, promote the establishment of an immunoscore for NSCLC. Such immunoscore should take into consideration a dynamic threshold for CD8^+^ TILs based on the expression of PD-L1 in neoplastic cells and PD-1 on immune cells. Such approach may be applied to different tumors, including pediatric ones such as metastatic neuroblastoma, known to express PD-L1 (26), for which the current therapies are largely unsuccessful. Given the important interaction between the PD-1/PD-L1 immune axis and CD8^+^ cell function, further studies aiming at better defining the predictive role of TILs for immunotherapy are needed.

## Data Availability Statement

The raw data supporting the conclusions of this article will be made available by the authors, upon reasonable request.

## Ethics Statement

The study was reviewed and approved by IRCCS Sacro Cuore Don Calabria Hospital Ethics Committee. Written informed consent for participation was not required for this study in accordance with the national legislation and the institutional requirements.

## Author Contributions

Conceptualization: EM and GB. Data curation: EM, GB, PB, and GQ. Formal analysis: EM, GB, GL, FC, MM, and AT. Funding acquisition: LM and GZ. Investigation: EM and GB. Methodology: EM, GB, MB, and GM. Project administration: EM and GB Resources: GB and GZ. Software: EM. Supervision: GZ and GB. Validation: AC, GR, AE, and GN. Roles/Writing - original draft: EM and GB. Writing – review and editing: LM, PV, NT, LQ, PB, and GN. All authors contributed to the article and approved the submitted version.

## Funding

This study was partially supported by Associazione Italiana per la Ricerca sul Cancro (id 21147 and 19920).

## Conflict of Interest

FC is a consultant and member of the scientific advisory board of TRIBVN Healthcare, France.

The remaining authors declare that the research was conducted in the absence of any commercial or financial relationships that could be construed as a potential conflict of interest.

## Supplementary Material

Supplementary Figure 1 Kaplan-Meier curves for DFS and OS based on CD8^+^ TILs using the 25^th^ percentile threshold **(A, B)** and 75^th^ percentile threshold **(C, D)**, considering the entire NSCLC cohort.

Supplementary Figure 2 Kaplan-Meier curves for DFS and OS based on CD8^+^ TILs using the median threshold in cases with tumors lacking PD-1^+^ TILs **(A, B)** or with PD-1^+^ TILs **(C, D)**.

Supplementary Figure 3 Kaplan-Meier curves for DFS **(A)** and OS **(B)** based on CD8^+^ TILs using the median threshold in PD-L1 negative tumors lacking PD-1^+^ TILs
