# Supplementary figures and images for: Impact of PD-L1 and PD-1 Expression on the Prognostic Significance of CD8+ Tumor-Infiltrating Lymphocytes in Non-Small Cell Lung Cancer
Source: Front Immunol. 2021 May 26;12:680973. doi: 10.3389/fimmu.2021.680973 (PMC8187779; doi:10.3389/fimmu.2021.680973)

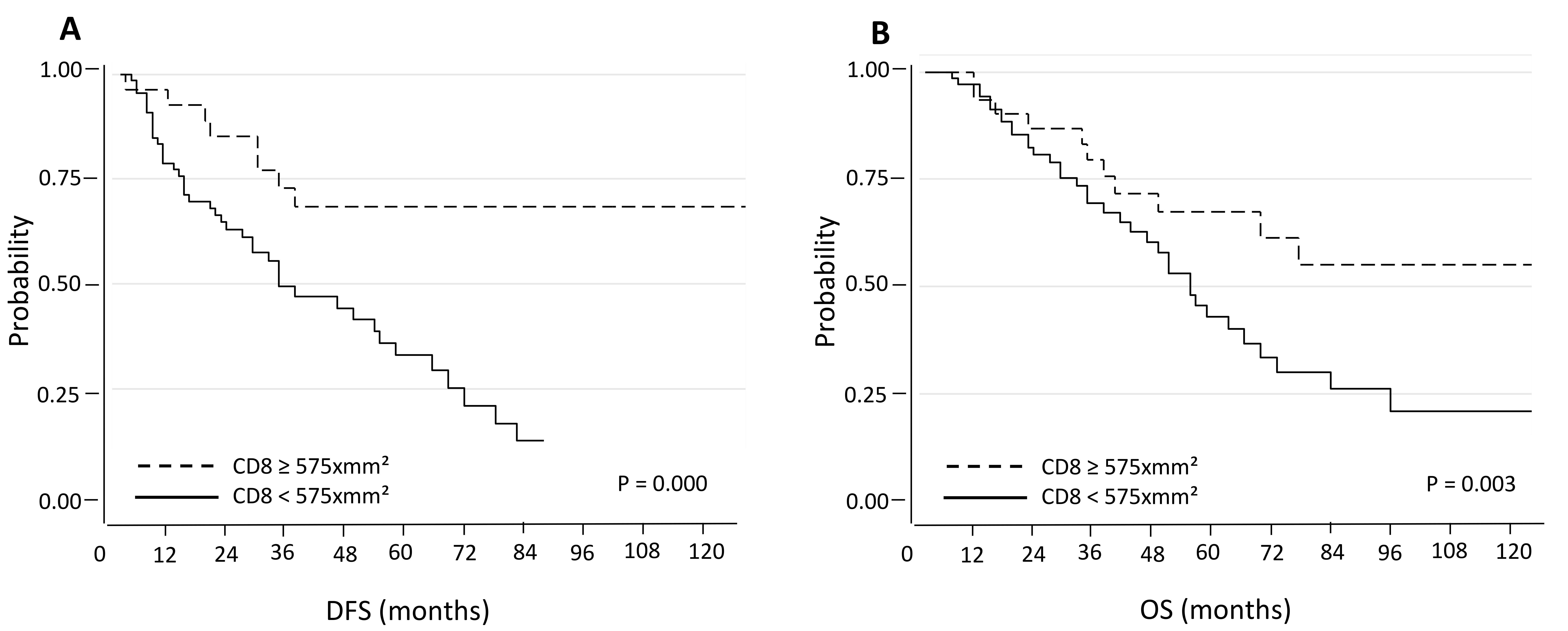

Supplement: Supplementary Figure 3 — Kaplan-Meier curves for DFS (A) and OS (B) based on CD8+ TILs using the median threshold in PD-L1 negative tumors lacking PD-1+ TILs [file Image_3.jpeg]
