# Supplementary material for: Impact of PD-L1 and PD-1 Expression on the Prognostic Significance of CD8+ Tumor-Infiltrating Lymphocytes in Non-Small Cell Lung Cancer
Source: Front Immunol. 2021 May 26;12:680973. doi: 10.3389/fimmu.2021.680973 (PMC8187779; doi:10.3389/fimmu.2021.680973)
Supplement: Supplementary file 4 [file Table_1.docx]

**Supplementary table 1:** associations between PD-L1 tumor expression and PD-1^+^ TILs

|  |  | **PDL1** | |  |
| --- | --- | --- | --- | --- |
|  | **Overall (%)** | **< 1 (%)** | **≥ 1 (%)** | **P value** |
| **Patients** | 314 | 208 | 106 |  |
| **PD-1 (7.5% threshold)** |  |  |  |  |
| Negative | 143 (45.6) | 105 (50.5) | 38 (35.9) | *0.023* |
| Positive | 154 (49.0) | 94 (45.2) | 60 (56.6) |  |
| Unknow | 17 (5.4) | 9 (4.3) | 8 (7.5) |  |
